# An Appeal for the Selection of a Proper Meat and Milk Inspector

**Published:** 1895-08

**Authors:** Otto Noack

**Affiliations:** Reading, Pa.


### SELECTIONS.

### AN APPEAL FOR THE SELECTION OF A PROPER MEAT AND MILK INSPECTOR.[1]

BY OTTO NOACK, V.S.,
OF READING, PA.

By an act of the Legislature of this State the Board of Health is given power to employ a dairy and food commissioner who shall inspect only meat and milk and prohibit the selling of diseased meat or impure milk. Such an inspector should be a trained veterinarian. The candidates for this position should be reported to the Department of Agriculture, and on the recommendation of this bureau the candidate should be appointed. No man should obtain this responsible position who is unable to represent the veterinary profession. How necessary such an inspector is your honorable body can see by the following figures, and if you only consider how many diseases are transferred to mankind by diseased meat you will appreciate the necessity of employing a competent man.

The following are the figures. Condemned animals unfit for food in only one abattoir in New York from July 1, 1892 to January 1, 1893: of 3601 cows slaughtered 492 were diseased, or 13.66 per cent. Of 492 diseased, 237 were affected with diseases dangerous to mankind (tuberculosis, anthrax, etc ), the other 255 were sick and not fit for food. Reports of the Bureau of Animal Industry will show what percentage of cows diseased by tuberculosis are found in the abattoir: Baltimore, 2.5 per cent. to 3.5 per cent. (a very favorable figure); New York, maximum to 98 per cent., minimum to 5 per cent. State Hospital in Norristown, of 183 animals, 135 were found tuberculous.

In Germany we find that slaughtered animals are affected: cows, 6.9 per cent.; oxen, 3.6 per cent.: steers, 2.6 per cent. Leipsic reached these numbers: cows, 26 per cent.; oxen, 19.5 per cent.; steers, 15 per cent.; calves, 9.3 per cent. In Am-

[1] Addressed to the Members of Select and Common Council of Reading, Pa.

sterdam, 1891, of 23,392 animals slaughtered, 1248, or 5.2 per cent., were tuberculous; 1892, of 25,454 animals shaughtered, 1332, or 5.3 per cent., were tuberculous; 1893, of 28,342 animals slaughtered, 1491, or 5 26 per cent, were tuberculous. Professor Eggeling (Berlin) reports that out of 37 cows 31 were tuberculous.

How easily this disease has been transferable to mankind you will see by the following. (*a*) Diseased through milk: Case reported by Dr. Stang, of Amorback; four infants in the hospital of Berne, by Dr. Demme; one case in North Hadley, Mass.; Dr. E. O. Shakespeare (*Med. News*, March 26, 1892), attributes one-fifth of all deaths in infants and young children, feeding on milk, to tuberculosis commencing in the digestive organs. (J. Law.) (*b*) Through meat: Tsherining, of Copenhagen, attended a veterinarian who had cut his finger in making a post-mortem examination of a tuberculous cow; the wound healed, but there remained a swelling which soon ulcerated and refused to heal, so that the whole tumefied mass had to be cut out; the microscope revealed the tuberculous process and the presence of the tubercle bacillus. Pfeiffer attended a Weimer veterinarian, of the name of Moses, thirty-four years old, of a good constitution, who in 1885 cut his right thumb deeply in making a post-mortem examination of a tuberculous cow; the wound healed, but six months later the cicatrix remained swollen, and in the autumn, 1886, the man had pulmonary tuberculosis with bacilli in his sputa; death occurred in two and one-half years after the wound; post-mortem examination revealed tuberculosis of the joint of the wounded thumb and in the lungs extensive tubercles and vomicæ. To Tsherining's case may be added the case of a young veterinary friend of the writer, who was inoculated in the hand in opening a tuberculous cow, and suffered from a tumefaction of the resulting cicatrix, with distinct tubercle bacilli; the surgical removal of the tumefaction manifestly saved the subject from a generalized tuberculosis. (J. Law, professor in Cornell University).

The danger and suffering that diseased pork can give humanity the following will show: 1. Cysticerces (cause of tapeworms). 2. Trichinosis. In Germany, found in the various abattoirs, pork-meat, 0.05–0.06 per cent. trichinous. According to Eulenberg, in American pork, 4 per cent., and Billings found, in Boston, of 2701 hogs killed, 154 or 5.7 per cent. trichinous. In Thuringia and neighborhood, where no meat inspection was made, epidemics in Heberslaben and Hettsbadt, 334 people sick (Kratz); Braunschweig, 1882, 254 people sick (Blasius); Halbersbadt, 1883, 250 people sick (Brouardel); Oberuinewalde, 1888, 235 people sick (Riedel); in Hanover, three epidemics in 1864–65, and 300 persons infected; since 1886 inspection, one epidemic, 50 persons affected by one hog not examined (Gerlach). In Illinois, 1893, many cases; in Pennsylvania, 1885–95, around Erie, over 50 cases found by Dr. Ed. W. Germer; in 1894, 2 cases in Lancaster (State Board of Health). Zenkel, of Dresden, reports a typhus epidemic, and after dissection of some corpses, the physicians did not find a trace of typhus, but found the muscles full of trichinæ.

I hope these examples will convince you of the necessity of employing a meat and milk inspector. I will add to it the recommendation of such an inspection of Assistant Surgeon-General W. C. W. Glazier (1880): "*Veterinary Supervision for Animals.* Such a supervision should be exercised both before and after slaughter. In small towns where there is no veterinary surgeon persons should be instructed by official veterinary surgeons, to whom all doubtful questions should be referred."

The appointed inspector's duties shall be to inspect: 1. All meat market-houses (fish, game, etc.). 2. Slaughter-houses and imported meat. The inspector shall be under the control of Board of Health, and report there all cases of condemned meat and milk. This law being enforced our city will follow other large cities in advancement, and have power to prevent the sale of all diseased food.

---

The recent unjust and unwarranted criticism of the veterinarians of the Philadelphia Horse Show, at certain decisions as to the soundness of animals exhibited, will, we trust, lead to a more severe exercise of this feature of such exhibitions in the future. Unsound and weak animals are not the kind we want to encourage the breeding of. The many years of thoughtless breeding, sacrificing size, conformation, color, and every other good quality to speed, or some other single point, has done enough injury in this intelligent age. The ill-built, shad-bellied, narrow-chested race of trotting-horses that have to be booted and weighted from the feet to the bodies, and still knock their limbs to pieces in their flights of speed, are a sad comment on our intelligence as breeders.

